# Prognostic impact of metabolic syndrome in patients with primary endometrial cancer: a retrospective bicentric study

**DOI:** 10.1007/s00432-024-05699-1

**Published:** 2024-04-03

**Authors:** Ina Shehaj, Slavomir Krajnak, Morva Tahmasbi Rad, Bahar Gasimli, Annette Hasenburg, Thomas Karn, Marcus Schmidt, Volker Müller, Sven Becker, Khayal Gasimli

**Affiliations:** 1 Department of Gynaecology and Obstetrics, University Medical Center of the Johannes Gutenberg University Mainz, Langenbeckstr. 1, 55131 Mainz, Germany; 2 Department of Gynaecology and Obstetrics, Johann Wolfgang Goethe University, Frankfurt Am Main, Germany; 3 Department of Gynaecology and Obstetrics, Jung-Stilling Hospital, Siegen, Germany

**Keywords:** Endometrial cancer, Metabolic syndrome, Survival, Obesity

## Abstract

**Purpose:**

Endometrial cancer (EC) is the most common gynaecological cancer. Its incidence has been rising over the years with ageing and increased obesity of the high-income countries’ populations. Metabolic syndrome (MetS) has been suggested to be associated with EC. The aim of this study was to assess whether MetS has a significant impact on oncological outcome in patients with EC.

**Methods:**

This retrospective study included patients treated for EC between January 2010 and December 2020 in two referral oncological centers. Obesity, arterial hypertension (AH) and diabetes mellitus (DM) were criteria for the definition of MetS. The impact of MetS on progression free survival (PFS) and overall survival (OS) was assessed with log-rank test and Cox regression analyses.

**Results:**

Among the 415 patients with a median age of 64, 38 (9.2%) fulfilled the criteria for MetS. The median follow-up time was 43 months.

Patients suffering from MetS did not show any significant differences regarding PFS (36.0 vs. 40.0 months, HR: 1.49, 95% CI 0.79–2.80 P = 0.210) and OS (38.0 vs. 43.0 months, HR: 1.66, 95% CI 0.97–2.87, P = 0.063) compared to patients without MetS. Patients with obesity alone had a significantly shorter median PFS compared to patients without obesity (34.5 vs. 44.0 months, P = 0.029). AH and DM separately had no significant impact on PFS or OS (p > 0.05).

**Conclusion:**

In our analysis, MetS in patients with EC was not associated with impaired oncological outcome. However, our findings show that obesity itself is an important comorbidity associated with significantly reduced PFS.

## Introduction

With approximately 382,000 new cases annually worldwide, endometrial cancer (EC) represents the most prevalent gynaecological malignancy in industrialized countries (Sung et al. 2021). More than 80% of patients with EC present with the International Federation of Gynecology and Obstetrics (FIGO) early stages (I-II) and have a 5-year overall survival (OS) rate of 95% (Raglan et al. 2019). However, the incidence rates have increased since the late 1990s in most developed countries (Sung et al. 2021). Metabolic diseases, including obesity (body mass index (BMI) > 30 kg/m^2^), and diabetes mellitus (DM), belong to the most important contributing factors for the rising rates (Raglan et al. 2019). Although unopposed estrogen exposure is considered a major driver of endometrial carcinogenesis, other additional factors such as chronic inflammation, insulin resistance, and hyperinsulinemia are allied to substantial risk factors for EC (Perez-Martin et al. 2022; Siegel et al. 2021). Previous epidemiological studies revealed that obesity, DM, and metabolic syndrome (MetS) are each linked to an increase in EC incidence, but their separate and combined influence on survival remains unclear (Raglan et al. 2019; Perez-Martin et al. 2022; Romanos-Nanclares et al. 2023; Morice et al. 2016). Conversely, Tang et al. described in a meta-analysis the perceived protective effects of metformin intake leading to a decrease in incidence rates and an improvement in OS in EC Patients. (Tang et al. 2017). Metformin disrupts cancer cell metabolism via direct inhibition of mitochondrial respiration, the mammalian target of rapamycin (mTOR), and the phosphoinositide 3-kinase (PI3K)/protein kinase B (AKT) pathways in EC cells (Arcidiacono et al. 2012; Yin et al. 2014; Zhao et al. 2018). In a case-control study conducted by Rosato et al., cancer risk was significantly increased for subjects with MetS (HR: 8.40, 95% CI 3.95–17.87) (Rosato et al. 2011). The authors showed that the most strongly associated factors with EC included a BMI >30 kg/m^2^ and meeting at least two criteria of AH, DM, and/or hyperlipidemia. Despite the strong relation between metabolic disease and EC, sufficient data about the impact of MetS on survival rates in patients with EC are lacking (Luo et al. 2014; Bjorge et al. 2010; Bing et al. 2022). This study aims to investigate the prognostic role of MetS in primary EC in order to provide evidence for cancer prevention and adjuvant treatment strategies.

## Materials and methods

For this retrospective analysis, we identified patients with primary EC from both gynaecological centers who underwent primary surgical therapy between January 2010 and December 2020. Clinicopathological data, treatment details, and follow-up information were obtained from the oncological registry, archives, and medical reports as of April 2022. The cohort was divided into two groups: patients with MetS and those without. Patients in the MetS group met the following criteria: obesity (defined as a body mass index (BMI) ≥30.0 kg/m^2^), DM, and AH requiring drug therapy. These patients were identified after screening clinical data for MetS. Both groups were compared regarding clinicopathological characteristics and survival data. Insufficient clinical information, particularly unclear MetS status, the diagnosis of synchronous malignant tumors, and severe internal diseases such as renal failure and/or liver cirrhosis, were defined as exclusion criteria. The classification of disease stage was based on the FIGO system, which was revised in 2008 (Pecorelli 2009; Soslow et al. 2019). Due to the increasing use of minimally invasive surgery in EC, most patients were treated via laparoscopy, with particularly frequent usage in the later years of the study. All surgical procedures were performed by or under the assistance of specialized gynecologic oncologists from both centers following established international guidelines. Preoperative BMI and patient comorbidities were recorded upon admission to the clinic. Conventional histology and immunohistochemical methods were performed and the results were confirmed by two specialized gynecological pathologists at our centers. Regular implementation of next-generation sequencing (NGS) and determination of protein p53 and mismatch repair protein (MMRP) deficiency were not standard during the study period.

After surgical treatment, all patients were discussed at a multidisciplinary tumor board (MTB), and adjuvant approaches were recorded in the MTB protocols. Following completion of primary therapy, patients underwent follow-up every three months for the first three years or when symptoms appeared. Routine follow-up care included clinical and sonographic examinations, and if recurrence was suspected, additional imaging examinations such as CT scans, MRI scans, or rarely PET-CT scans were conducted. The survival intervals progression free survival (PFS) and overall survival (OS) were defined as the time from the date of surgical treatment to the date of histological confirmation of cancer recurrence and death or the date of the last follow-up, respectively. Written informed consent was not required due to the retrospective nature of this study. This analysis was conducted within the framework of the UCT-19-2021 project and received approval from the Institutional Review Boards of the UCT and the Ethical Committee at the University Hospital Frankfurt.

### Statistical analysis

Statistical analyses were conducted using IBM SPSS Version 27.0 statistical software package (SPSS Inc., Chicago, IL, USA). Nonparametric survival functions, including Kaplan–Meier curves and the log-rank test, were employed to determine outcome probabilities. A Chi-square test was used to compare categorical clinicopathological parameters between patients with and without MetS. All statistical tests were two-sided, and a p-value < 0.05 was deemed significant. Univariate Cox regression was conducted to identify independent prognostic factors for PFS and OS in all patients. This analysis was also performed separately for patients with and without Metabolic Syndrome (MetS). Variables that demonstrated statistical significance were subsequently verified through multivariable analysis, incorporating the variables summarized in Table 2 and 3.

## Results

**Fig. 1:**
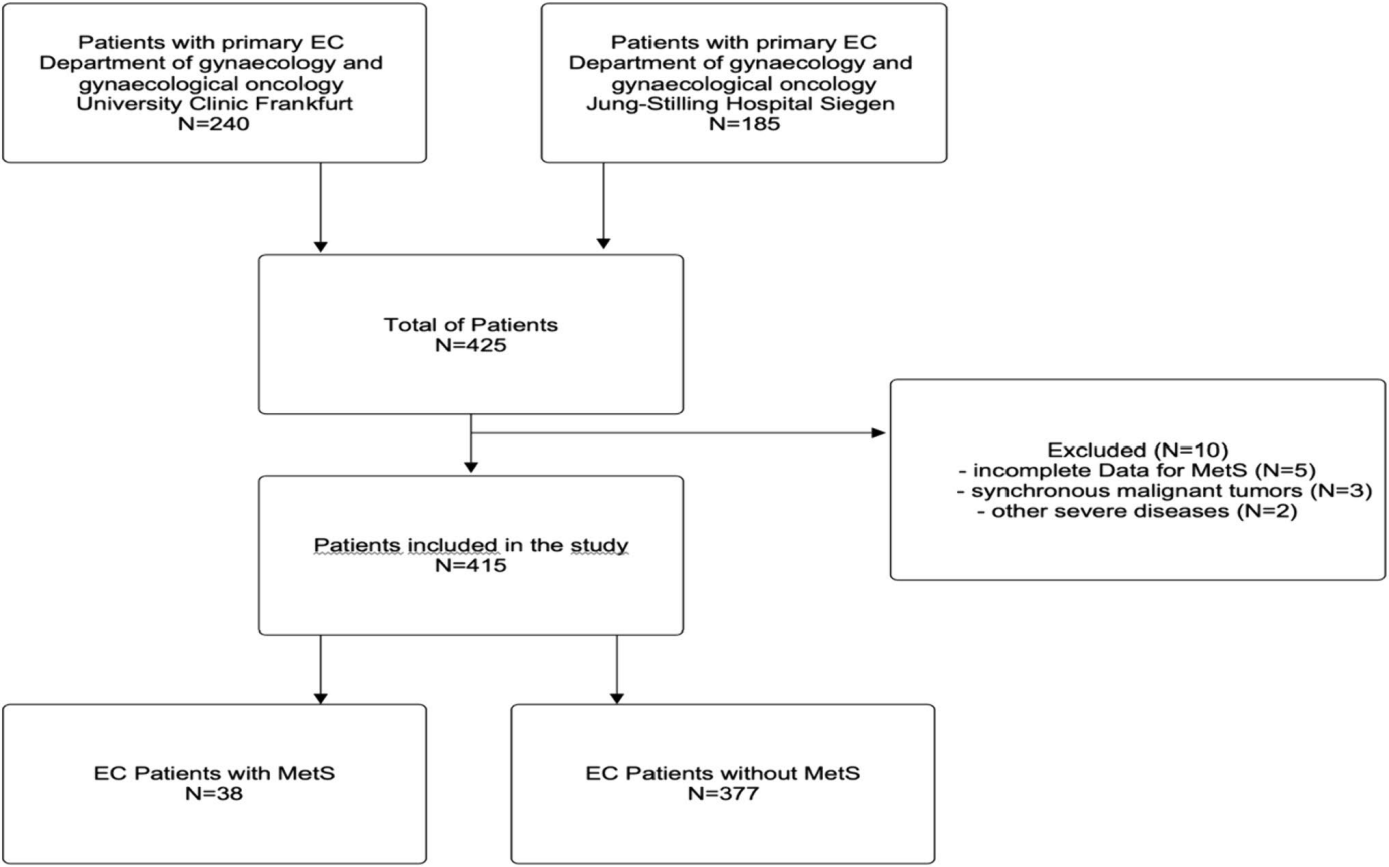
Flowchart of patients’ selection for analysis

A total of 415 patients with primary EC participated in this retrospective analysis (Fig. 1).

The majority of the study's participants (80.7%) were postmenopausal, with a median age of 64 years (range: 28–91) for the entire cohort. The patients were categorized into two groups based on the presence of metabolic syndrome: the MetS group and the non-MetS group. The MetS group were significantly older compared to the non-MetS group (70.5 vs. 63.0 years; p = 0.001). The characteristics of the patients are shown in (Table 1).

**Table 1:** Comparison of baseline clinicopathological data of patients with EC by MetS

Variables	All patients total n = 415, (%)	With MetS N %) total n = 38, (9.2%)	Without MetS N (%) total n = 377, (90.8%)	P value
Demographic characteristics				
Age, median (range) years	64.0 (28.0–91.0)	70,5 (40.0–91.0)	63.0 (28.0–91.0)	0.001
BMI, mean (range) kg/m^2^	28.0 (18.0–72.0)	33.0 (30.0–60.0)	27,52 (18.0–72.0)	0.001
ECOG score				
0	225 (54.2)	17 (44.7)	208 (55.2)	0.421
1	120 (28.9)	14 (36.8)	106 (28.1)	
2	37 (8.9)	6 (15.8)	31 (8.2)	
3	12 (2.9)	1 (2.6)	11 (2.9)	
4	3 (0.7)	0	3 (0.8)	
Unknown	18	0	18 (4.8)	
Diabetes mellitus type 2	70 (16.9)	38	32 (8.5)	< 0.001
Arterial hypertension	183 (44.1)	38	145 (38.5)	< 0.001
Clinical-pathological tumor parameters				
Tumor stage (FIGO)				
IA	191 (46)	15 (39.5)	176 (46.7)	0.617
IB	103 (24,8)	12 (31.6)	91 (24.1)	
II	36 (8.7)	4 (10.5)	32 (8.5)	
IIIA	10 2.4)	0 (0)	10 (2.7)	
IIIB	14 (3.4)	3 (7.9)	11 (2.9)	
IIIC	20 (4.8)	2 (5.3)	18 (4.8)	
IVA	4 (1.0)	0 (0)	4 (1.1)	
IVB	29 (7.0)	2 (5.3)	27 (7.2)	
Missing	8 (1.9)	0 (0)	8 (2.1)	
Histological subtype				
EEC	383 (92.3)	37 (97.4)	346 (91.8)	0.370
Non-EEC	32 (7.7)	1 (2.6)	31 (8.2)	
Histological grading				
G1	205 (49.4)	16 (42.1)	189 (50.1)	0.149
G2	116 (28.0)	16 (42.1)	100 (26.5)	
G3	85 (20.5)	6 (15.8)	79 (21)	
Missing	9 (2.2)	0 (0)	9 (2.4)	
Lymph nodes				
N0	315 (75.9)	30 (78.9)	285 (75.6)	0.330
N1	36 (8.7)	5 (13.2)	31 (8.2)	
N2	7 (1.7)	1 (2.6)	6 (1.6)	
Missing	57 (13.7)	2 (5.3)	55 (14.6)	
Surgical approach				
Minimally invasive	228 (54.9)	20 (52.6)	208 (55.2)	0.271
Vaginal	12 (2.9)	3 (7.9)	9 (2.4)	
Laparotomy	156 (37.6)	15 (39.5)	141 (37.4)	
No surgical therapy	15 (3.6)	0 (0)	15 (4.0)	
Missing	4 (1.0)	0 (0)	4 (1.1)	
Lymphadenectomy				
Pelvic lymphadenectomy	132 (31.8)	11 (28.9)	121 (32.1)	0.715
Paraaortic lymphadenectomy	87 (21.0)	7 (18.4)	80 (19.1)	0.683
Pelvic and paraaortic lymphadenectomy	87 (21.0)	7 (18.4)	80 (19.1)	0.683
Adjuvant treatment				
None	296 (71.3)	26 (68.4)	270(71.6)	
Radiotherapy	119 (28.7)	14 (31.6)	107 (28.4)	0.919
EBRT	9 (2.2)	1 (2.6)	8 (2.1)	
VBT	94 (22.7)	10 (26.3)	84 (22.3)	
ERBT + VBT	16 (3.9)	1 (2.6)	15 (4.0)	
Chemotherapy	77 (18.6)	3 (7.9)	74 (19.6)	0.093
Mortality				
Recurrence	89 (21.4)	11 (28.9)	78 (20.7)	0.490
Mortality	111 26.7)	15 (39.5)	96 (25.5)	0.542

*EC* endometrial cancer, *MetS* metabolic syndrome, *CI* confidence interval, *HR* hazard ratio, *BMI* body mass index, *ECOG* Eastern Cooperative Oncology Group, *FIGO* Fédération Internationale de Gynécologie et d'Obstétrique, *EEC* endometrioid endometrial cancer *N1* pelvic lymph node metastasis, *N2* paraaortic lymph node metastasis, *EBRT* external beam radiation therapy, *VBT* vaginal brachytherapy, *OS* overall survival, *PFS* progression-free survival, p-value < 0.05 is considered to be significant

The most prevalent comorbidities included arterial hypertension (44.1%), diabetes mellitus type 2 (16.9%), and obesity (41.2%), with a median BMI of 28.0 kg/m^2^. As a result, 9.2% of the cases met the criteria for MetS. The majority of patients (83.1%) had a favorable ECOG performance score (ECOG 0/1). The endometrioid histological subtype (92.3%) was the most common, followed by serous (5.1%), mixed serous-endometrioid (1.4%), and clear cell (1.2%) EC. High-grade differentiation (G2/G3) of the tumor was observed in 201 cases (48.5%), with a significant proportion (70.8%) of them being in the early stages of the disease (FIGO IA and B). Pelvic and para-aortic lymph node metastasis, as well as distant metastasis, were identified in 36 cases (8.7%), 7 cases (1.7%), and 33 cases (8%), respectively. Adjuvant chemotherapy was more frequently administered to the non-MetS group than the MetS group (19.6% vs. 7.9%, p = 0.093), despite similar advanced tumor stage distributions in both groups. However, statistical significance was not achieved. One possible explanation could be that patients in the MetS group, who often had comorbidities and were older, were less inclined to receive chemotherapy. All patients underwent hysterectomy and bilateral salpingo-oophorectomy (BSO), with 37.6% undergoing laparotomy and 54.9% receiving minimally invasive surgery.

A total of one hundred and thirty-two patients (31.8%) underwent pelvic lymph node dissection, out of which eighty-seven (21.0%) had pelvic and para-aortic lymphadenectomy. No clinically significant differences in the outcome were observed in patients with or without pelvic (p = 0.715) and paraaortic lymphadenectomy (p = 0.683) between the MetS and non-MetS groups. Among those who underwent lymphadenectomy in the MetS group, five (13.2%) had pathologic pelvic lymph nodes, and one (2.6%) had pathologic paraaortic lymph nodes.

Despite the specific challenges, laparoscopy was performed in most patients with obesity. The majority (52.6%) of patients in the MetS group received minimally invasive surgery, while 39.5% underwent laparotomy. Interestingly, there were no significant differences in the choice of surgical approach between both groups (p = 0.271). In the comparative analysis between the MetS and non-MetS groups, significant differences were not observed in clinicopathological factors, including ECOG score (p = 0.421), FIGO stage (p = 0.617), histological subtype (p = 0.370), histological grading (p = 0.149), lymph node involvement (p = 0.330), surgical approaches (p = 0.271), and adjuvant treatment.

### Association between metabolic syndrome and survival

The median follow-up time was 43 months, ranging from 1 to 120 months. We compared the survival data between patients with MetS and those without MetS. The primary goal of this study was to determine the effect of MetS and its individual components on the PFS of patients with EC. The comparative analyses of the groups revealed no statistically significant differences in terms of overall survival (HR: 1.66, 95% CI 0.965–2.869, p = 0.063) and recurrence-free survival (HR: 1.49, 95% CI 0.792–2.801, p = 0.210), as shown in (Table 2).

**Table 2:** Univariate and multivariate analysis of metabolic syndrome and its components associated with prognosis (OS, overall survival; PFS, progression-free survival) in patients with endometrial cancer

Variables	Univariate OS HR (95% CI)	P value	Multivariate OS HR (95% CI)	P value	Univariate PFS HR (95% CI)	P Value	Multivariate PFS HR (95% CI)	P value
Obesity	1.21 (0.83–1.78)	0.323			1.21 (0.83–1.78)	0.029	1.02 (1.002–1.047)	0.029
Arterial hypertension	1.13 (0.78–1.64)	0.515			1.03 (0.68–1.57)	0.896		
Diabetes mellitus	1.45 (0.93–2.27)	0.099			1.11 (0.64–1.90)	0.714		
Metabolic syndrome	1.66 (0.97–2.87)	0.063			1.50 (0.79–2.80)	0.210		

*CI* confidence interval, *HR* hazard ratio, *OS* overall survival, *PFS* progression-free survival, p-value < 0.05 is considered to be significant

**Fig. 2:**
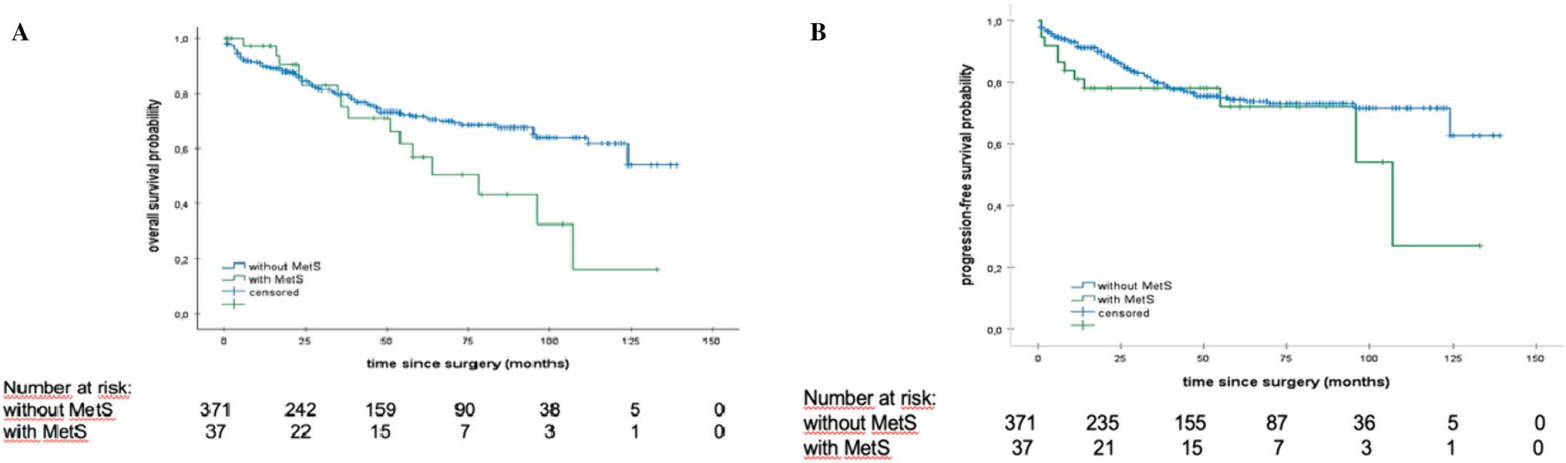
**A** Kaplan–Meier analyses of OS regarding the presence of metabolic syndrome Patients with MetS versus without MetS, median OS: 38.0 versus 43.0 months, log rank: p = 0.063. **B** Kaplan–Meier analyses of PFS regarding the presence of metabolic syndrome Patients with MetS versus without MetS, median PFS: 36.0 versus 40.0 months, log rank: p = 0.210

The PFS for patients with MetS versus patients without MetS was 36.0 months versus 40.0 months, respectively (p = 0.210). Similarly, OS rates were worse for patients with MetS compared to patients without MetS: 38.0 months versus 43.0 months, respectively (p = 0.063) (Fig. 2).

However, there was a significant correlation between obesity and PFS. No significant correlation was observed regarding obesity and OS (HR: 1.213, 95% CI 0.826–1.781, p = 0.323) (Table 2).

**Fig. 3:**
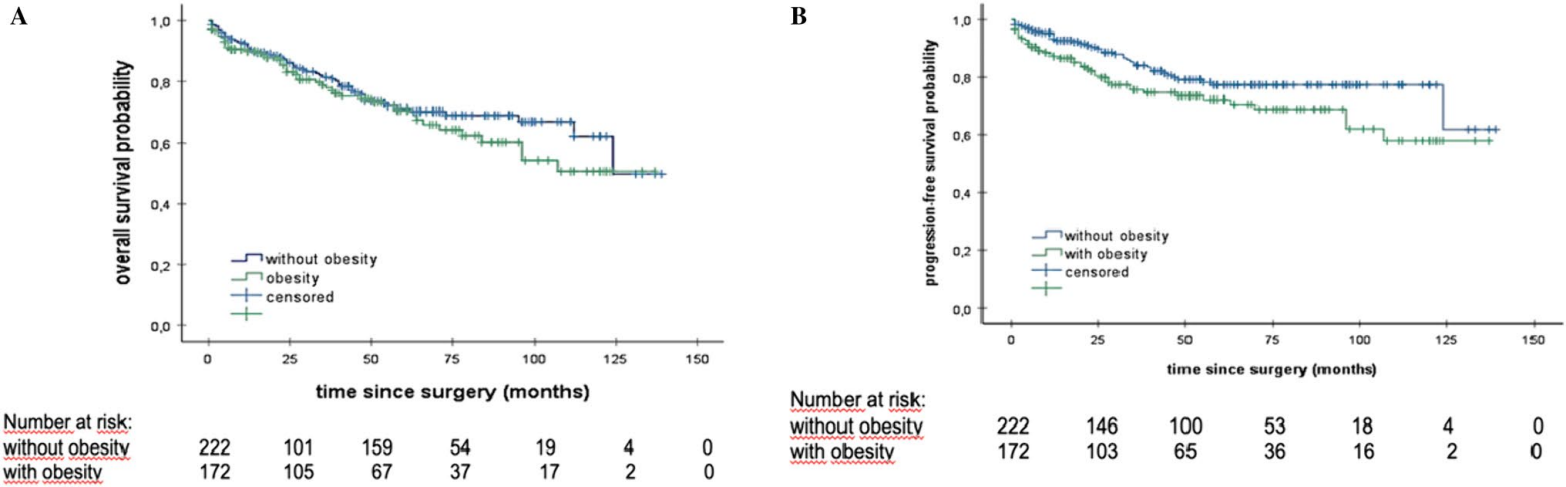
Kaplan–Meier analyses of OS and PFS regarding the presence of obesity. **A** Patients with obesity versus patients without obesity, median OS: 38.0 vs. 46.0 months, p = 0.323 **B** Patients with obesity versus patients without obesity, median PFS: 34.5 vs. 44.0 months log rank: p = 0.029

For patients with obesity alone PFS was significantly reduced compared to the cohort without obesity (34.5 vs. 44.0 months, HR: 1.606; 95% CI 1.043–2.472, p = 0.029) (Fig. 3).

To evaluate “the dose reponse” relation of BMI in our patients to the survival we classified this study cohort regarding BMI in three subgroups: obesity class 1(BMI 30–35 kg/m^2^), obesity class 2 (BMI 35–40 kg/m^2^) and obesity class 3 (BMI > 40). In the univariate analysis for all patients included in the study but also just for the patients with MetS, no “dose reponse” relation could be shown regarding OS (p > 0.05) and PFS (p > 0.05). The range of BMI could not be identified as an indipendent factor related to OS and PFS.

Our observations did not reveal statistically significant differences in terms of shorter OS (p > 0.05) or poorer PFS (p > 0.05) in patients with isolated arterial hypertension (AH) or diabetes mellitus type 2 (DM) (Table 2).

Tables 3 and 4 present the results of Cox regression univariate and multivariate analysis for the risk of recurrence in all patients and in patients with MetS, respectively.

**Table 3:** Univariate and multivariate analysis of factors associated with prognosis (OS, overall survival; PFS, progression-free survival) in patients with endometrial cancer

Variables	Univariate OS HR (95% CI)	P value	Multivariate OS HR (95% CI)	P value	Univariate PFS HR (95% CI)	P value	Multivariate PFS HR (95% CI)	P value
Patient age (years)	1.05 (1.03–1.06)	< 0.001	1.04 (1.02–1.06)	< 0.001	1.03 (1.01–1.05)	< 0.001	1.02 (1.002–1.047)	0.029
Mean BMI (kg/m^2^)	0.99 (0.97–1.01)	0.331			1.0 (0.97–1.02)	0.693		
ECOG Score	1.83 (1.50–2.22)	< 0.001	1.5 (1.04–1.84)	0.024	1.64 (1.31–2.05)	< 0.001	1.53 (1.155–2.021)	0.003
Histological subtype	1.584 (1.19–2.10)	0.007	0.64 (0.41–1.01)	0.054	1.69 (1.28–2.25)	0.002	0.62 (0.39–0.99)	0.049
Histological grade of differentiation	2.50 (1.97–3.20)	< 0.001	1.66 (1.21–2.27)	0.002	2.93 (2.23–3.85)	< 0.001	1.89 (1.34–2.67)	< 0.001
FIGO Stage	1.97 (1.68–2.32)	< 0.001	1.49 (1.22–1.84)	< 0.001	2.02 (1.70–2.41)	< 0.001	1.45 (1.17–1.81)	< 0.001
Lymphadenectomy	1.18 (1.01–1.38)	0.041	1.13 (0.94–1.37)	0.197	0.99 (0.81–1.21)	0.901		
Pelvic lymphadenectomy	1.36 (1.02–1.80)	0.043	1.17 (0.95–1.46)	0.142	1.24 (0.93–1.66)	0.142		
Paraaortic lymphadenectomy	1.12 (0.85–1.48)	0.402			1.01 (0.78–1.32)	0.921		
LVSI	2.26 (1.58–3.21)	< 0.001	0.84 (0.44–1.61)	0.621	2.63 (1.88–3.66)	< 0.001	0.75 (0.37–1.52)	0.425
Surgery (laparoscopic/laparotomy)	1.81 (1.38–2.37)	< 0.001	0.50 (0.32–0.79)	0.003	1.52 (1.12–2.07)	0.011	0.67 (0.42–1.07)	0.09
Chemotherapy	3.56 (2.37–5.34)	< 0.001	1.88 (1.12–3.16)	0.017	5.81 (3.77–8.98)	< 0.001	3.20 (1.26–4.09)	< 0.001
Radiotherapy	1.07 (0.69–1.67)	0.762			1.50 (0.95–2.35)	0.082		

*EC* endometrial cancer, *CI* confidence interval, *HR* hazard ratio, *BMI* body mass index, *ECOG* Eastern Cooperative Oncology Group, *FIGO* Fédération Internationale de Gynécologie et d'Obstétrique *LNM* lymph node metastasis LVSI lymph-vascular invasion *OS* overall survival, *PFS* progression-free survival, p-value < 0.05 is considered to be significant

**Table 4:** Univariate and multivariate analysis of factors associated with prognosis (OS, overall survival; PFS, progression-free survival) in EC-Patients with MetS

Variables	Univariate OS HR (95% CI)	P value	Multivariate OS HR (95% CI)	P value	Univariate PFS HR (95% CI)	P value	Multivariate PFS HR (95% CI)	P value
Patient age (years)	1.02 (0.97–1.07)	0.459			1.08 (1.00–1.16)	0.041	1.13 (1.04–1.22)	0.002
Mean BMI (kg/m^2^)	1.00 (0.95–1.06)	0.942			0.95 (0.88–1.03)	0.212		
ECOG Score	1.12 (0.66–2.18)	0.811			0.81 (0.36–1.81)	0.613		
Histological subtype	1.71 (0.834–3.50)	0.223			0.36 (0.00–1.47)	0.491		
Histological grade of differentiation	1.43 (0.91–2.24)	0.148			1.63 (1.05–2.54)	0.030	1.18 (0.74–1.89)	0.500
FIGO Stage	1.82 (1.03–3.22)	0.049			2.33 (1.32–4.11)	0.003	4.67 (1.91–11.39)	< 0.001
Lymphadenectomyomy	1.34 (0.82–2.38)	0.272			0.98 (0.42–2.23)	0.951		
Pelvic lymphadenectomy	1.74 (0.56–5.41)	0.347			2.19 (0.59–8.17)	0.258		
Paraaortic lymphadenectomy	2.43 (0.73–8.11)	0.153			1.99 (0.50–7.98)	0.351		
LVSI	0.71 (0.34–4.90)	0.711			1.56 (0.39–6.26)	0.542		
Surgery (laparoscopic vs. laparotomy)	1.21 (0.58–2.55)	0.611			0.73 (0.29–1.88)	0.510		
Chemotherapy	2.96 (0.60–14.7)	0.182			0.24 (0.05–1.18)	0.077		
Radiotherapy	0.92 (0.23–3.07)	0.911			1.38 (0.34–5.51)	0.651		

*EC* endometrial cancer, *CI* confidence interval, *HR* hazard ratio, *BMI* body mass index, *ECOG* Eastern Cooperative Oncology Group, *FIGO* Fédération Internationale de Gynécologie et d'Obstétrique *LNM* lymph node metastasis LVSI lymph-vascular invasion *OS* overall survival, *PFS* progression-free survival, p-value < 0.05 is considered to be significant

In the univariate analysis for all patients, the significant variables affecting PFS were age, ECOG Score, FIGO stage, tumor grade, histological subtype, type of surgery and chemotherapy (p < 0.05) (Table 3). In the univariate analysis for the MetS Group, age, histological grade of differentiation, and FIGO-Stage were associated with poorer PFS (all p < 0.05), whereas FIGO-Stage alone was associated with worse OS (p = 0.05) (Table 4).

In the multivariate Cox regression analysis, we found that the following factors retained their prognostic significance for PFS in patients with MetS: age (HR 1.13; 95% CI 1.04–1.22, p = 0.002) and FIGO-Stage (HR 4.67; 95% CI 1.91–11.39, p < 0.001).

## Discussion

In this retrospective study, we observed no oncological impact of MetS in patients with primary EC. However, obesity alone represents an important comorbidity associated with a worse PFS in the present series. The reason might be associated with the number and characteristics of the population.

The prognostic significance of MetS and its components on EC has been previously explored by few studies.

In particular, epidemiological and preclinical studies have shown that the pathogenesis of endometrioid EC is closely related to estrogen (Zhao et al. 2018; Yin et al. 2019; Mitsuhashi et al. 2014). These pathophysiological changes may explain the role of MetS in endometrial carcinogenesis (Bjorge et al. 2010).

The metabolic tumor microenvironments formed in MetS are closely involved in the development of EC via several potential mechanisms (Kyo and Nakayama 2020). Since the production of estrogen is an intermediate product of lipid metabolism, abnormal lipid metabolism has an influence on the secretion of estrogen and the balance of estrogen and progesterone (Li et al. 2023; Palmisano et al. 2017; Rochlani et al. 2017). Especially in patients with obesity, a hyper-estrogenic state caused by the presence of the aromatase enzyme in adipose tissue is identified, which catalyzes the conversion of androgens to estrogen in postmenopausal women (Byers and Sedjo 2015). In addition, MetS is associated with chronic insulin resistance, which can lead to the overproduction of reactive oxygen species and contribute to DNA damage (Arcidiacono et al. 2012). Hyperestrogenism and hyperglycemia, associated with obesity and metabolic syndrome, play pivotal roles in cancer pathogenesis. They stimulate cell proliferation and angiogenesis through distinct mechanisms and induce hyperplasia in endometrial tissue.

Other published meta-analyses and cohort studies support a relationship between MetS components such as diabetes, obesity, and hypertension and an increased risk of EC (Luo et al. 2014; Bing et al. 2022; Modesitt et al. 2012).

Our present analysis seeks to investigate the prognostic significance of individual and combined components of MetS in patients with EC. According to our knowledge, there have been limited reports on the prognostic effect of MetS in patients with EC (Li et al. 2023; Kokts-Porietis et al. 2020; Yang et al. 2021). Controversial data have been published regarding survival data in EC related to the presence of MetS (Rosato et al. 2011). Additionally, there is a lack of unified criteria for MetS (Balkau and Charles 1999). Kokts-Porietis et al. conducted a prospective cohort study with 540 patients with EC, of which 325 had MetS at diagnosis. They reported that MetS and an elevated waist circumference (≥88 cm) were associated with worse OS for EC (HR: 1.98, 95% CI 1.07–3.67, HR: 2.12, 95% CI 1.18–3.80, respectively) (Kokts-Porietis et al. 2020).

Similiarly, Yang et al. retrospectively analyzed outcomes of 506 patients with EC diagnosed between 2010 and 2016, among whom 153 (31%) were diagnosed with MetS (Yang et al. 2021). Their results indicated that MetS was closely related to OS (HR: 2.14, 95% CI 1.07–4.28, P = 0.032) and PFS (HR: 1.80, 95% CI 1.0–3.3, P = 0.045) in EC patients. The OS decreased in patients with ≥ 3 components compared to those with 1–2 or 0 components (p = 0.045), while no apparent difference was observed for PFS rates (p = 0.069). After adjusting for other variables, including age, histological type, tumor grade, and stage, MetS was not associated with the prognosis of EC.

Similar to the report by Yang et al., the results of our retrospective study showed that MetS in patients with EC was not associated with a worse oncological outcome. However, a trend toward significance was noticed regarding the OS (p = 0.063). We could show in our study, that the patients with MetS were significantly older than the patients without MetS. However, no statistically significant impact of age on OS could be verified in the Cox regression analysis.

Additionally, we could confirm that obesity alone remains an important comorbidity in patients with EC associated with a worse PFS. In the multivariable analysis, age and FIGO Stage were associated with worse PFS in patients with MetS.

Since 2013, we have witnessed a significant shift in the diagnosis and treatment of EC. Following the The Cancer Genome Atlas (TCGA) research network's announcement of the four new molecular subtypes of EC, numerous societies (e.g. World Health Organization, the International Society of Gynecological Pathologists, European Society of Gynaecological Oncology/European Society for Radiotherapy and Oncology/European Society of Pathology) have embraced these classifications and aligned their guidelines with the evolving understanding of disease development (Concin et al. 2021). An updated evaluation of risk stratification, along with more consistent adjuvant therapy concepts, is anticipated in the forthcoming ESGO/ESTRO/ESP guidelines (Concin et al. 2021). This promises to deliver risk-adapted therapies to patients, reducing the likelihood of under- and over-treatment, and thereby enhancing prognostic outcomes, while also alleviating the financial burden on healthcare systems.

The potential limitations of this study include the retrospective design, which encompasses some missing data, a small number of enrolled patients with heterogeneity of patient and tumor characteristics, as well as the lack of information about the molecular profile. However, the inclusion of well-documented cases and the performance of surgeries and pathological reviews by the same experienced team at our clinics may enhance the importance of our results.

In conclusion, Metabolic Syndrome (MetS) could not be established as a prognostic factor for primary EC; however, obesity significantly reduced survival rates. These findings support the notion that modifying lifestyle factors and reducing obesity-related risk factors through dietary changes and regular exercise as preventive measures may not only decrease the risk of cancer but also reduce the mortality rate among patients with EC. Further research is needed to correlate the prognostic significance of molecular profiles with MetS and obesity. This could help clinicians to better predict the risk of recurrence and death in patients with EC and such accompanying comorbidities.

## Data Availability

The datasets generated during the current study are available from the corresponding author on reasonable request.
